# Cerebrospinal fluid kappa free light chains as biomarker in multiple sclerosis—from diagnosis to prediction of disease activity

**DOI:** 10.1007/s10354-022-00912-7

**Published:** 2022-02-08

**Authors:** Harald Hegen, Klaus Berek, Florian Deisenhammer

**Affiliations:** Department of Neurology, Medical University of Innsbruck, Anichstr. 35, 6020 Innsbruck, Austria

**Keywords:** Clinically isolated syndrome, FLC index, Oligoclonal bands, Progression, Relapse

## Abstract

Multiple sclerosis (MS) is a chronic immune-mediated disorder of the central nervous system that shows a high interindividual heterogeneity, which frequently poses challenges regarding diagnosis and prediction of disease activity. In this context, evidence of intrathecal inflammation provides an important information and might be captured by kappa free light chains (κ-FLC) in the cerebrospinal fluid (CSF). In this review, we provide an overview on what is currently known about κ‑FLC, its historical development, the available assays and current evidence on its diagnostic and prognostic value in MS. Briefly, intrathecal κ‑FLC synthesis reaches similar diagnostic accuracy compared to the well-established CSF-restricted oligoclonal bands (OCB) to identify patients with MS, and recent studies even depict its value for prediction of early MS disease activity. Furthermore, detection of κ‑FLC has significant methodological advantages in comparison to OCB detection.

## Introduction

Multiple sclerosis (MS) is a chronic inflammatory immune-mediated disease of the central nervous system (CNS) that mainly affects young adults and bears the risk of physical and cognitive disability [1].

Diagnosis of MS requires the combination of clinical signs and symptoms with paraclinical findings obtained by magnetic resonance imaging (MRI) and cerebrospinal fluid (CSF) analysis [2]. Evidence of intrathecal immunoglobulin G (IgG) synthesis in the CSF, although not specific for MS, increases diagnostic certainty in the appropriate clinical setting [3] and substitutes for dissemination in time according to current diagnostic criteria [2].

Besides establishing MS diagnosis, one of the main challenges for neurologists counselling patients with MS is weighing benefits versus risks of certain disease-modifying therapies (DMTs) [4]. An ever-increasing number of DMTs have been proven to reduce the number of relapses, accumulation of disability and brain MRI activity [5] and current treatment concepts recognize the importance of early treatment towards suppressing disease activity below the level of detectability [6]. However, the interindividual courses of MS are extremely variable [7] and there is also a certain risk for treatment-associated adverse events. Since criteria guiding decisions when to start treatment in early MS and, in case, whether to choose a moderately or a highly efficacious DMT are still controversially debated, there is an urgent need of biomarkers to predict disease activity [4, 8]. So far, the number of brain MRI lesions and the presence of intrathecal IgG synthesis in the CSF imply some prognostic value [9].

As depicted above, the value of CSF analysis for diagnosis of MS and for prediction of disease activity after the first demyelinating CNS event is unquestioned. However, for the detection of intrathecal IgG synthesis as a marker for intrathecal B cell activity, several different laboratory methods have been developed in the last half century. Quantitative methods that require the measurement of IgG concentrations in CSF and serum followed by calculation of certain formulae such as IgG index [10], Reiber [11] or Auer & Hegen formulae [12] referring patient’s individual values to a predefined upper normal limit are mainly hampered by their low sensitivity. The detection of oligoclonal IgG bands (OCB) by isoelectric focusing (IEF) followed by immunoblotting is nowadays the gold standard. This technique compares paired CSF and blood samples of each individual patient. Intrathecal IgG synthesis is present if OCB are present in CSF without corresponding bands in serum [13]. It ensures a high diagnostic sensitivity and specificity both of approximately 90% [14]. However, this method enables only a qualitative determination of intrathecal IgG synthesis (i.e., returns either a positive or a negative result), is technically demanding, time-consuming, costly and rater-dependent [13].

## Kappa free light chains in the CSF as an emerging biomarker

**Fig. 1:**
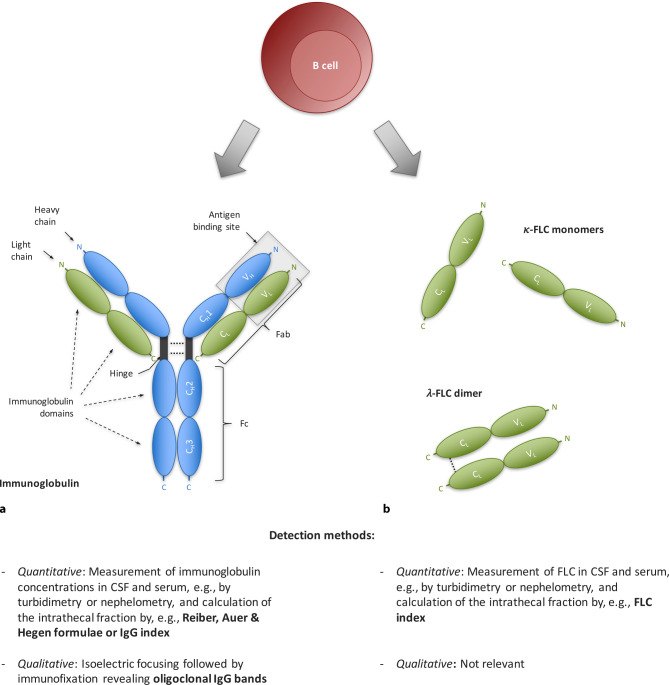
Schematic illustration of the molecular structure of immunoglobulins and free light chains. B cells produce (**a**) intact immunoglobulins and (**b**) in excess free light chains. Both immunoglobulins and FLC serve as biomarker for B cell activity. **a** Immunoglobulins consist of two identical heavy chains (*blue*) and two identical light chains (*green*). Each heavy chain consists of four immunoglobulin domains linked by a hinge region. Differences in the structure of the constant regions (C_H_1, C_H_2 and C_H_3) determine the isotype (IgG, M, A, D, E) and subclass of the immunoglobulin (e.g., IgG1–4), while the variable domain (V_H_) contributes to the antigen binding site. Each light chain consists of two immunoglobulin domains. Differences in the structure of the constant region (C_L_) determine the isotype of free light chain (either κ or λ), while the variable domain (V_L_) contributes to the antigen binding site. Accordingly, both the heavy chains and light chains form the amino-terminal variable (V) regions responsible for antigen recognition; the carboxyl-terminal constant (C) regions mediate effector functions. **b** Free light chains show the same structure as light chains bound within the intact immunoglobulin. FLC have a molecular weight of approximately 24 kD and consist of the two immunoglobulin domains C_L_ and V_L_. Differences in the structure of the constant region (C_L_) determine the isotype of the free light chain (either κ or λ). Whereas κ‑FLC mainly exist in the form of monomers, λ‑FLC are present as covalent dimers. *C*_*H*_ constant heavy chain domain, *C*_*L*_ constant light chain domain, *Fab* fragment antibody binding, *Fc* fragment crystallisable, *FLC* free light chain, *V*_*H*_ variable heavy chain domain, *V*_*L*_ variable light chain domain

Besides intact immunoglobulins that consist of light chains and heavy chains bound together via disulfide bonds and noncovalent interactions [15], B cells also produce light chains in 10–40% excess over heavy chains and secrete them as free forms into the blood circulation [16]. These free light chains (FLC) have a molecular weight of approximately 24 kD and consists of two immunoglobulin domains, a constant region that specifies the isotype of free light chain (either κ or λ) and a variable domain (Fig. 1; [15]). If bound, the variable light chain domain is part of the immunoglobulin antigen binding site; the function in the free forms is not fully elucidated [16]. κ‑FLC exist mainly in the form of monomers, whereas λ‑FLC are present as covalent dimers [16]. In the last few years, a multitude of studies have highlighted the value of κ‑FLC in CSF as another biomarker—instead of immunoglobulins—for intrathecal B cell activity in patients with MS, not least due to significant methodological advantages.

## History of FLC detection

FLCs were discovered more than 150 years ago, when in 1847 Henry Bence Jones described a protein in the urine of a patient with severe bone pain and fractures that precipitated upon addition of nitric acid [17]. The so-called Bence Jones proteins evolved to an important diagnostic marker for patients with multiple myeloma. More than 100 years after its discovery, the Bence Jones protein was identified as monoclonal FLC [18]. Developments in laboratory methods brought up protein electrophoresis and immunofixation electrophoresis; however, these methods still had limited sensitivity so that low level FLC under physiological or oligosecretory conditions, e.g., in immune-mediated diseases, were not detectable, and allowed only qualitative determination [18]. Attempts to quantify FLC were initially hindered by difficulties of producing antibodies specific to FLC that do not cross-react with light chains bound in intact immunoglobulins. The breakthrough was achieved in 2001 by Bradwell and coworkers who dissociated light chains from heavy chains and then raised antibodies directed against unique epitopes on FLC that are normally “hidden” in the conformational structure of an intact immunoglobulin [19]. These anti-human FLC-specific antibodies could then be used to develop assays that exclusively detect FLC at least a hundred times more sensitive than previous methods with detection limits down to approximately 1 mg/L. Nowadays, FLC can be measured in serum as well as in CSF by use of two types of detection antibodies: either polyclonal [19] or monoclonal [20] detection antibodies.

## Elevated κ-FLC in the CSF of patients with MS

κ‑FLC in the CSF—similar to immunoglobulins or other proteins—originate either from blood by diffusion across the blood–CSF barrier or are produced within the intrathecal compartment under pathological conditions [21]. Conceptually, it seems necessary to determine the locally synthesized κ‑FLC fraction separate from the blood-derived fraction (as it is also done for IgG). Most studies calculated the κ‑FLC index that considers the CSF/serum albumin quotient (Q_alb_) which is an established marker for the blood–CSF barrier function [22] and corrects for the absolute serum κ‑FLC level. The κ‑FLC index is determined by following formula [23, 24]:

$$\kappa -FLC\,\textit{index}=\frac{\kappa -\mathrm{FLC}_{\mathrm{CSF}}/\kappa -\mathrm{FLC}_{\text{Serum}}}{Q_{\mathrm{alb}}}$$

It has been consistently shown that the κ‑FLC index reaches a high diagnostic accuracy to identify patients with MS. An overview of current evidence—retrieved in a systematic literature search [23–40]—is provided in Table 1. For the κ‑FLC index, diagnostic sensitivity ranges from 52 to 98% (weighted average: 87%) and specificity ranges from 68 to 100% (weighted average: 89%). For OCB, sensitivity ranges from 37 to 100% (weighted average: 84%) and specificity from 74 to 100% (weighted average: 90%). The reported sensitivity of OCB is in accordance with a previous meta-analysis [41]. Applying a difference-in-differences model showed that the mean difference of diagnostic sensitivity between κ‑FLC index and OCB was +2% and of specificity was −2%, i.e., overall the diagnostic accuracy of κ‑FLC index and OCB was equal.

**Table 1:** Diagnostic value of κ‑free light chain index in patients with multiple sclerosis

Reference	Type of controls^a^	No. of control subjects	No. of MS patients	McDonald criteria	Laboratory method	κ‑FLC index cut-off	Elevated κ‑FLC index in MS, *n*	Sensitivity, %	Normal κ‑FLC index in controls, *n*	Specificity, %	OCB positive in MS, *n*	Sensitivity, %	OCB negative in controls, *n*	Specificity, %
[25]	NIND/IND/PIND	1149	75	2010	Ne/N Latex	9.58	69	92	1115	97	71	95	1072	93
[26]		299	146	2010/2017	Tu/Freelite	5.8	76	52	282	94	54	37	299	100
[28]		197	45	2017		6.6	42	93	172	87	40	89	179	91
[29]	NIND/IND	105	71	Not specified	Ne/N Latex	12.3	68	96	105	100	65	92	99	94
[30]		85	37	Not specified	Ne/Freelite	5.9	28	76	77	91	33	89	69	81
[31]		253	67	2010		10.463	58	87	193	76	63	94	187	74
[33]		83	59	Not specified	Tu/Freelite	12.45	46	78	64	77	46	78	66	80
[32]		258	127	2017	Ne/N Latex	5.0	122	96	208	81	123	97	214	83
[24]		219	284	2005/2010	Tu/Freelite	6.6	264	93	181	83	245	86	202	92
[34]		42	34	2017		9.4	32	94	29	68	34	100	38	90
[35]		240	133	2017	Ne/N Latex	5.0	124	93	205	85	127	96	204	85
[27]		456	84	2017	Tu/Freelite	6.2	75	89	383	85	71	85	405	89
[36]	NIND	368	41	2001/2005	Ne/Freelite	5.9	40	98	318	86	39	95	338	92
[23]		60	60	2005		5.9	56	93	57	95	56	93	59	98
[37]		97	96	2010	T/Freelite	7.5	87	91	88	91	79	82	91	94
[40]		30	68	2017	N/Freelite	3.09	49	72	30	100	38	56	30	100
[38]		50	80	2010	N/N Latex	5.3	77	96	48	96	73	91	49	98
[39]	HC/SC	60	62	2010		7.15	56	90	60	100	54	87	60	100

A search of the electronic database PubMed was performed on November 17, 2021 using the terms “multiple sclerosis” and “free light chains” and limited to the time period between January 1, 2005 and November 17, 2021. Titles and abstracts of identified articles written in English were screened and the full text of potentially relevant articles were assessed for inclusion criteria. Studies were included if they were original articles investigating the diagnostic value of κ‑FLC index in patients with MS compared to other neurological diseases and used nephelometry/turbidimetry for κ‑FLC measurement. *κ-FLC* kappa free light chain, *OCB* oligoclonal bands

Following original articles were included: [25] Senel 2019, [26] Ferraro 2020, Eur J Neurol, [28] Sanz Diaz 2021, [29] Pieri 2017, [30] Valencia-Vera 2018, [31] Gurtner 2018, [33] Bayart 2018, [32] Crespi 2019, [24] Leurs 2020, [34] Gudowska-Sawczuk 2020, [35] Vecchio 2020, [27] Ferraro 2020, Diagnostics (Basel), [36] Presslauer 2008, [23] Presslauer 2016, [37] Christiansen 2018, [40] Altinier 2019, [38] Emersic 2019 and [39] Duell 2020.

The diagnostic value of κ‑FLC index and OCB was compared by a difference-in-differences model. Therefore, for each study, the difference of diagnostic sensitivity of κ‑FLC index and OCB (∆sensitivity), as well as the difference of diagnostic specificity of κ‑FLC index and OCB (∆specificity) was calculated. Then, also the sum of ∆sensitivity and ∆specificity was calculated (∆overall). Finally, the mean of all ∆values was calculated. This statistical analysis revealed a mean ∆sensitivity of 2%, ∆specificity of −2%, and ∆overall of 0%. This result indicate that there is no difference in the diagnostic performance of κ‑FLC index and OCB to discriminate patients with MS from controls

*HC* healthy controls, *IND* inflammatory neurological disease controls (other than MS), *MS* multiple sclerosis, *Ne* Nephelometry, *NIND* non-inflammatory neurological disease controls, *PIND* peripheral inflammatory neurological disease controls, *SC* symptomatic controls, *Tu* Turbidimetry, *κ‑FLC* kappa free light chain, *OCB* oligoclonal bands

^a^Control population of studies were labelled/categorized according to the “Consensus definitions and application guidelines for control groups in cerebrospinal fluid biomarker studies in multiple sclerosis” [42]

The wide range of diagnostic sensitivity and specificity for both the κ‑FLC index and OCB arises from a certain heterogeneity between studies. It is evident that specificity of κ‑FLC index is lowered when patients with inflammatory neurological disease (IND) were included into the control group. κ‑FLC in CSF are—similar to CSF-restricted OCB—a sign of intrathecal inflammation and thus can support the diagnosis of MS, but they are not specific for MS. The spectrum of diseases which show intrathecal κ‑FLC synthesis is probably identical to that with CSF-restricted OCB, even though studies on the frequency of intrathecal κ‑FLC synthesis in other neurological disease are still rare. Apart from a mixture of different IND as part of control populations (Table 1) that had κ‑FLC synthesis in up to 32%, dedicated disease-specific studies exist only for a few entities, e.g., neuroborreliosis [43, 44].

## κ-FLC index associated with early MS disease activity

There are only a few studies on the predictive value of the κ‑FLC index in MS. An overview is given in Table 2. These studies reported that the presence of intrathecal κ‑FLC synthesis is associated with conversion from CIS to MS [45–49] and that the κ‑FLC index predicted the time to conversion to MS as well as disability progression [49, 50]. However, these studies had some methodological limitations. A multivariate approach that considers other already known risk factors especially MRI activity is critical to identify the independent prognostic effect of the κ‑FLC index and to weigh its impact on the outcome.

**Table 2:** Prognostic value of κ‑free light chain index in patients with multiple sclerosis

Ref	Age (years) mean ±SD	Fem. (%)	McDonald criteria	OCB (%)	FU (months) median	End- point	Patients reaching endpoint					Patients not reaching endpoint					Assay	Cut-off κ‑FLC index	Statistical analyses	Main findings
							No.	κ‑FLC index		OCB		No.	κ‑FLC index		OCB					
								Positive *N*	Sensitivity %	Positive *N*	Sensitivity %		Negative *N*	Specificity %	Negative *N*	Specificity %				
[51]	NA	NA	2001	NA	55 (mean)	Conv. to CDMS	24	10	42	NA	NA	0	–	–	–	–	Ne/Freelite	> 50	Mann Whitney U	Time to CDMS did not differ between patients with high (> 50) and low (< 50) κ‑FLC index
[48]	35 (min 15–max 62)	88	NA	62	>24	Conv. to CDMS	38	35	92	NA	NA	39	25	64	NA	NA	Ne/Freelite	> 10.62	Cox regression	κ‑FLC index predicted time to CDMS (HR 5.3)
[50]	34 ± 11	64	2017	92	47 (mean)	MSSS	NA	NA	NA	NA	NA	NA	NA	NA	NA	NA	Ne/ N latex	NA	Linear regression	κ‑FLC index predicted MSSS
[47]	42 ± 11	78	2010	NA	39	Conv. to MS^a^	12	12	100	NA	NA	11	3	27	NA	NA	Ne/Freelite	≥10.6	Cox regression	κ‑FLC index predicted time to MS (50% of patients with κ‑index ≥ 10.6 converting in 21 months)
[49]	30 ± 9	86	2017	82	79	EDSS progression^b^	18	NA	NA	17	94	10	NA	NA	4	40	Ne/Freelite	NA	Spearman correlation	κ‑FLC index correlated with shorter time to EDSS progression (r = −0.55)
						Conv. to CDMS	NA	NA	NA	NA	NA	NA	NA	NA	NA	NA				κ‑FLC index correlated with shorter time to CDMS (r = −0.59)
[52]	33 ± 10	68	2017	90	47	Conv. to CDMS	38	13	34	–	–	50	44	88	–	–	Ne/ N latex	> 100	Cox regression	κ‑FLC index predicted time to CDMS (11 vs. 36 months in patients with high [>100] vs. low [≤ 100] κ‑FLC index) Predictive value of κ‑FLC index was superior to that of OCB
								36	95	36	95		10	20	7	14		≥ 6.6		
						EDSS ≥ 3	8	2	25	–	–	78	61	78	–	–		> 100	Mann Whitney U	κ‑FLC index did not differ between patients reaching EDSS ≥ 3 or not at the end of follow-up
								7	88	8	100		11	14	9	12		≥ 6.6		

A search of the electronic database PubMed was performed on November 17, 2021 using the terms “multiple sclerosis” AND “free light chains” AND “prognosis”, “predict” or “conversion” limited to the time period between January 1, 2005 and November 17, 2021. Titles and abstracts of identified articles written in English were screened and the full text of potentially relevant articles were assessed for inclusion criteria. Studies were included if they were original articles investigating the prognostic value of κ‑FLC index in patients with clinically isolated syndrome in terms of various endpoints (e.g., conversion to MS) and used nephelometry/turbidimetry for κ‑FLC measurement. Following original articles were included: [51] Presslauer 2014, [48] Menéndez-Valladares 2015, [50] Vecchio 2019, [47] Gaetani 2020, [49] Salavisa 2020 and [52] Berek 2021

*CDMS* clinically definite multiple sclerosis, *Conv.* conversion, *EDSS* Expanded Disability Status Scale, *Fem.* females, *FLC* free light chain, *FU* follow-up, *HR* hazard ratio, *MSSS* Multiple Sclerosis Severity Score, *MS* multiple sclerosis, *N* nephelometry, *NA* not available, *No.* number, *OCB* oligoclonal bands, *ref* reference, *SD* standard deviation

^a^Conversion to MS was defined by clinical or radiological means

^b^EDSS progression was defined as an increase in EDSS score of ⩾ 1.5 points from a baseline EDSS score of 0, ⩾ 1.0 point from a baseline EDSS score of 1.0–5.5, or ⩾ 0.5 point from a baseline EDSS score ⩾ 6.0, confirmed after 6 months of follow-up

There is one recent study that fulfills these requirements providing class II evidence that in patients with early MS, high κ‑FLC index is an independent risk factor for early second clinical attack. A cohort of 88 patients with a first CNS demyelinating event (mostly monofocal, 45% myelitis, 30% optic neuritis, 24% affection of brainstem/cerebellum), at a mean age of 33 years and with a female predominance of 68% were followed over 4 years. In all, 38 (43%) patients converted to clinically definite MS (CDMS) within the observation period. In multivariate Cox regression analysis adjusting for age, sex, MRI lesion load and activity at baseline, administration of corticosteroids at baseline and DMT during follow-up revealed that κ‑FLC index predicts time to second clinical attack. This study showed that patients with κ‑FLC index > 100 at baseline had a twice as high probability for a second clinical attack within 12 months than patients with low κ‑FLC index; within 24 months, the chance in patients with high κ‑FLC index was 4 times as high as in patients with low κ‑FLC index. The median time to second attack was 11 months in patients with high κ‑FLC index, whereas 36 months in those with low κ‑FLC index [52].

## Advantages of κ-FLC index compared to OCB

Current evidence suggests that determination of κ‑FLC index has some advantages over OCB detection. Even though it seems that there is no relevant difference with regard to diagnostic accuracy (Table 1), κ‑FLC can be easily measured by nephelometry which is—in contrast to the detection of OCB—a reliable, labor-saving and cost-efficient method [20]. Moreover, κ‑FLC index returns a metric result covering a range from approximately 1 up to 500 [23], i.e., it is a quantitative parameter, while OCB status is dichotomous returning either a positive or negative result as assessed by visual inspection [13]. The advantage of a metric result seems important especially for predicting disease activity. In the most recent study on the predictive value of κ‑FLC index—as previously mentioned [52]—which included patients with a first CNS demyelinating event, OCB were detected in 95% of patients who converted to CDMS during the 4‑year follow-up (CDMS converters), whereas non-converters were OCB positive also in 86% of cases. As a continuous variable, κ‑FLC index overcame the weak performance of OCB by further stratification. κ‑FLC index also significantly differed between OCB-positive CDMS converters and OCB-positive nonconverters and predicted CDMS conversion also within the cohort of OCB-positive patients [52]. Despite these promising results and clear methodological advantages of κ‑FLC index over OCB, the latter is still considered the gold standard. Before κ‑FLC index might be introduced into clinical routine, a few issues still need to be clarified, e.g., whether calculation of intrathecal κ‑FLC synthesis is superior to determination of absolute κ‑FLC concentrations in CSF or which cut-off should be applied. These two open issues are discussed in the following.

## Open issues

### Determining κ-FLC index or absolute CSF κ-FLC values

As mentioned above, one might argue that determining the locally synthesized fraction of κ‑FLC separate from the blood-derived fraction is necessary to capture an intrathecal inflammatory process. And indeed, the majority of studies used the κ‑FLC index (Table 1) or calculated an intrathecal κ‑FLC fraction by empirically determined Q_alb_-dependent reference limits [25, 43, 51, 53], whereas some studies included the CSF/serum κ‑FLC ratio (Q_κ‑FLC_) [30, 38, 54–58]. Other authors determined the absolute CSF κ‑FLC concentrations [31] arguing that the contribution of blood-derived FLC to the total CSF FLC concentration is low in cases with intrathecal synthesis. In fact, the intrathecal fraction of κ‑FLC is greater than 80% in most MS patients [23], and around 15% of CIS/MS patients showed even higher absolute κ‑FLC concentrations in CSF than in serum that proves an intrathecal synthesis per se [59]. To further elaborate this research question, a recent study separated patients into low and high CSF κ‑FLC categories (based on median values) and observed that CSF κ‑FLC concentration, Q_κ‑FLC_ and κ‑FLC index showed similar diagnostic performance in the high category, but not in the low category with inferiority of CSF κ‑FLC and to some extent also of Q_κ‑FLC_. This is in line with a previous study reporting that Q_FLC_ depends almost exclusively on the amount of intrathecally synthesized FLC in cases of intrathecal B cell activity (defined by presence of oligoclonal FLC bands), whereas a correlation of Q_alb_ and Q_FLC_ was observed in cases of absent intrathecal B cell activity (defined by negative oligoclonal FLC bands) [56]. Thus, there is evidence that the impact of serum κ‑FLC levels and Q_alb_ is negligible in patients with strong intrathecal κ‑FLC synthesis, but probably not in patients with only low or modest intrathecal κ‑FLC production. Further studies applying multivariate statistics are required to compare these different approaches.

### Establishing cut-off values

Before κ‑FLC index can be introduced into clinical routine, cut-offs have to be established. Different cut-off values might apply depending on the clinical question, e.g., to provide an upper reference limit as determined in a control population (either healthy or e.g. a symptomatic control [42]), to differentiate MS from other IND or to classify patients according to their risk for future disease activity. The so far published cut-off values differentiating MS from other neurological diseases ranged from 3.09 to 12.45 (Table 1). As κ‑FLC index values indeed vary between diseases with high values in MS, followed by other IND and then by non-IND [32, 35, 60], different cut-off values might be useful. For example, one study showed that patients with MS had κ‑FLC index of approximately 90, whereas patients with neuromyelitis optica spectrum disease that is relevant differential diagnosis of MS had values of roughly 20 and control patients values of 4 [60].

Studies that address reproducibility of κ‑FLC index using different assays, platforms and cut-offs between centers are needed, too. Although some work has already been performed in terms of absolute serum κ‑FLC concentrations, this is still lacking for κ‑FLC index. κ‑FLC index might show different robustness, as a ratio (of the CSF/serum κ‑FLC concentration, used for calculation of the κ‑FLC index) is usually less prone to laboratory variations.

## Conclusions

κ‑FLC are a promising biomarker that might replace OCB detection. With regard to its diagnostic value, κ‑FLC index shows a high accuracy similar to that of OCB, but has also significant methodological advantages as an easy, reliable, fast, labor- and cost-saving method. With regard to its prognostic value, the benefit could evolve—either stand alone or in combination with others—to identify early MS patients with a higher risk for further disease activity, e.g., shorter time to a second attack. These patients could be advised to start DMT early or use highly effective DMT, as there is evidence that the time to the second attack has a prognostic impact on long-term disability [61, 62] and that early treatment significantly delays conversion to CDMS as well as disability progression [63–65]. Conversely, there is a certain proportion of patients showing a mild disease course who may not need a potentially harmful, psychologically distressing and, last but not least, costly DMT.

Whereas the high diagnostic value is already supported by a multitude of studies, further studies are still required to replicate the independent prognostic value of κ‑FLC index in early MS. Apart from harmonization efforts as depicted above to establish a widely applicable cut-off to definite positivity, potential influential factors such as corticosteroid treatment [52, 66], DMT or different disease phases (relapse versus stable remission) on κ‑FLC index also have to be explored.

Thus, there is convincing evidence that κ‑FLC index reliably indicates intrathecal inflammation in MS, might replace OCB determination and probably takes us one step closer to tailored medicine in MS.
